# Deep learning-based smart speaker to confirm surgical sites for cataract surgeries: A pilot study

**DOI:** 10.1371/journal.pone.0231322

**Published:** 2020-04-09

**Authors:** Tae Keun Yoo, Ein Oh, Hong Kyu Kim, Ik Hee Ryu, In Sik Lee, Jung Sub Kim, Jin Kuk Kim

**Affiliations:** 1 Department of Ophthalmology, Aerospace Medical Center, Republic of Korea Air Force, Cheongju, South Korea; 2 Department of Anesthesiology and Pain Medicine, Seoul Women’s Hospital, Bucheon, South Korea; 3 Department of Ophthalmology, Dankook University Hospital, Dankook University College of Medicine, Cheonan, South Korea; 4 B&VIIt Eye Center, Seoul, South Korea; National University of Singapore, SINGAPORE

## Abstract

Wrong-site surgeries can occur due to the absence of an appropriate surgical time-out. However, during a time-out, surgical participants are unable to review the patient’s charts due to their aseptic hands. To improve the conditions in surgical time-outs, we introduce a deep learning-based smart speaker to confirm the surgical information prior to cataract surgeries. This pilot study utilized the publicly available audio vocabulary dataset and recorded audio data published by the authors. The audio clips of the target words, such as left, right, cataract, phacoemulsification, and intraocular lens, were selected to determine and confirm surgical information in the time-out speech. A deep convolutional neural network model was trained and implemented in the smart speaker that was developed using a mini development board and commercial speakerphone. To validate our model in the consecutive speeches during time-outs, we generated 200 time-out speeches for cataract surgeries by randomly selecting the surgical statuses of the surgical participants. After the training process, the deep learning model achieved an accuracy of 96.3% for the validation dataset of short-word audio clips. Our deep learning-based smart speaker achieved an accuracy of 93.5% for the 200 time-out speeches. The surgical and procedural accuracy was 100%. Additionally, on validating the deep learning model by using web-generated time-out speeches and video clips for general surgery, the model exhibited a robust and good performance. In this pilot study, the proposed deep learning-based smart speaker was able to successfully confirm the surgical information during the time-out speech. Future studies should focus on collecting real-world time-out data and automatically connecting the device to electronic health records. Adopting smart speaker-assisted time-out phases will improve the patients’ safety during cataract surgeries, particularly in relation to wrong-site surgeries.

## Introduction

Medical errors, such as wrong-site surgeries, can be significantly devastating patients as well as surgeons. Operating on an incorrect surgical site is the most common medical error [1]. Ophthalmic surgeries on the wrong eye could occur owing to the carelessness of surgical participants. According to previous reports, wrong-site surgeries still continue to occur in the field of ophthalmology [2]. Recent studies suggests that a preoperative discussion, known as a surgical time-out, can significantly assist in decreasing the risk of wrong-site surgeries [3]. During a surgical time-out, the surgical team can confirm the patient’s identity, surgical site, and name of the procedure. However, time-outs are not always conducted accurately, and surgical errors continue to occur [4].

Cataract surgery is the most frequently undertaken surgical procedure in developed societies [5]. Furthermore, patients suffering from age-related cataracts may have concomitant medical conditions that may increase the risk of medical errors. Considering a significant number of patients in ophthalmic clinics, without an appropriate time-out, wrong-site surgeries may occur as the surgeon may find it difficult to identify each surgical case. The final token time-out before cataract surgeries can be ineffective if the surgeon and other participants do not consider it important [6]. Therefore, a consistent use of a preoperative checklist is recommended to confirm the surgical information; however, in reality, the checklist is not used for every cataract surgery. Moreover, during a time-out, surgical participants are unable to review patient charts due to their aseptic hands.

Recently, artificial intelligence-based techniques have revolutionized many fields ranging from medical data analyses to intricate image classification [7,8]. These contributions are not limited to research as deep learning techniques and highly efficient hardware are being introduced in clinics as well [9]. In addition, deep learning has been applied to speech recognition and several commercial smart speakers that exhibit a reliable performance in capturing human voices [10]. Smart speakers can provide virtual assistants with hands-free and voice-only interaction for surgeons, who have to ensure that their hands remain aseptic during surgery. However, application of smart speakers in medical fields is limited due to technical difficulties. Recently, a research group demonstrated the use of a smart speaker for interventional radiology procedures [11]. This device could capture a human voice and provide information about the intervention device sizing. It showed a potential to assist surgeons during a sterile procedure. However, there has been no report of a smart system that can confirm surgical information using a time-out procedure to improve safety.

Here, we introduce a deep learning-based smart speaker to confirm the surgical information during a time-out. Especially, we focus on the accurate detection of the surgical site and confirm the surgical site by comparing it with the patient’s information before the start of the surgery. This paper presents a pilot study designed for the deep learning-based assessment of speech recognition during a time-out in a cataract surgery and the development of a smart speaker to assist with a hands-free time-out.

## Methods

### Overview

**Fig 1:**
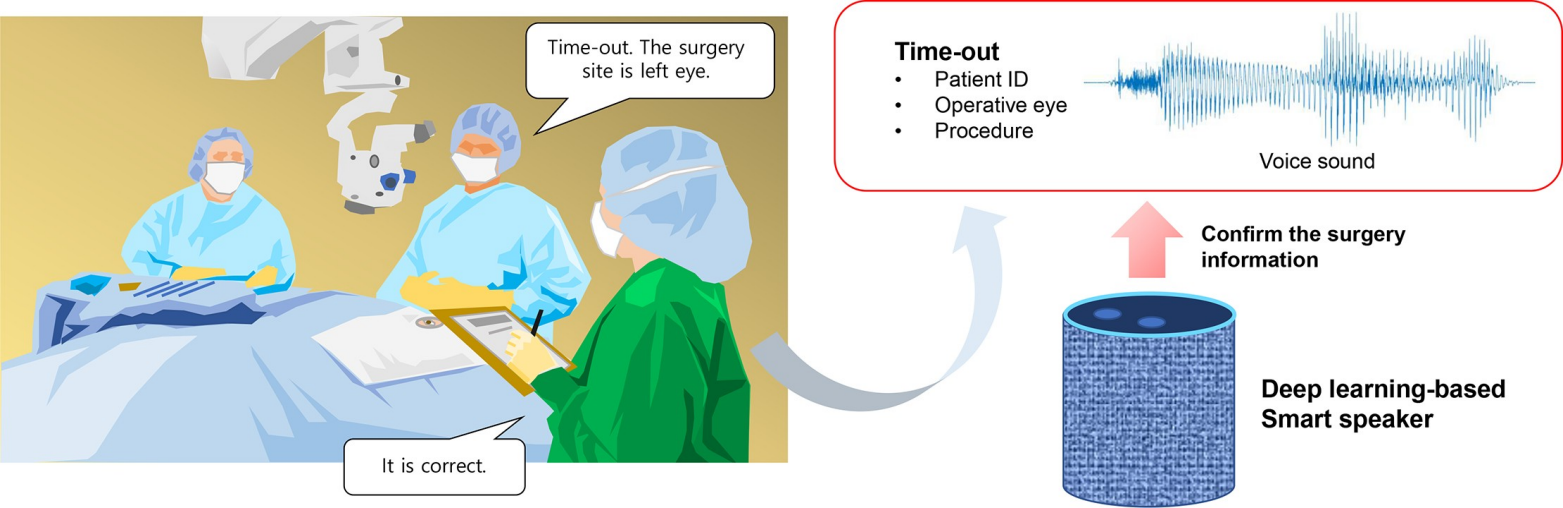
A deep learning-based smart speaker in ophthalmic surgery to confirm surgical information.

In this study, we designed a smart speaker that confirmed the surgical information based on a human voice. Fig 1 demonstrates the illustration of this proposed approach. The experimental process complied with the declaration of Helsinki. The approval of the ethics committee was not required because the researchers used a public database and voice data recorded by the authors to build the proposed deep learning model. There were no human or animal experiments undertaken in this pilot study. The Speech Commands dataset that was used in this study was collected by Google and released publicly, and it is available at the website https://www.tensorflow.org/tutorials/sequences/audio_recognition [12]. In addition, recorded voice and noise data are available as Mendeley Data repositories (http://dx.doi.org/10.17632/rwh74vrz8y).

### Training dataset

The dataset for the deep learning model is sourced from a short speech vocabulary that includes 16 target words, unknown words, and background noise. Detailed information about target word dataset is presented in Table 1. The Speech Commands dataset is chosen as the primary data for speech recognition [12]. This dataset consists of over 65,000, one-second audio recordings of more than 30 short words. The selected subset of the Speech Commands included the words *left*, *right*, *one*, *two*, *three*, *four*, *five*, *six*, *seven*, *eight*, *nine*, and *zero*. The remaining 18 words of the Speech Commands dataset, including *backward*, *bed*, *go*, *dog*, *tree*, *on*, and *learn*, were categorized as the “*unknown”* class. The *“background”* class consisted of one-second sound files that were randomly extracted from the background noise and silent sounds of the Speech Commands dataset. In the experiments, we attempted to add additional short words from various text-to-voice tools. Detailed information about the text-to-voice process is presented in S1 Fig. Researchers recorded the target words such as *time-out*, *cataract*, *phacoemulsification*, and *intraocular lens*, with varying accents, speed, and voice tones provided by the text-to-voice tools. As the voice interface relied on keyword spotting to initialize the interactions in most devices, “*time-out*” was assigned as a keyword to initialize the automated detection. Finally, a dataset that consists of the same word spoken by different people was compiled for training and validation. The Speech Commands dataset provides basic noise data including background sounds from white noise, pink noise, exercise, and doing the dishes. Additional noise from the operating room, including vital monitoring sound and background sound of surgery, were also added to the noise database. The noise recordings were captured using Samsung Galaxy S10 and AKG headphones.

**Table 1:** Sound dataset for the target words.

Words	N	Total size (MB)	Source	Purpose
**Time-out**	451	58.1	Recorded by the authors	Start time-out
**Right**	2,367	71.2	Open dataset [12]	Surgery site
**Left**	2,353	71.0	Open dataset [12]	Surgery site
**One**	2,370	71.1	Open dataset [12]	Patient ID
**Two**	2,373	71.3	Open dataset [12]	Patient ID
**Three**	2,356	70.9	Open dataset [12]	Patient ID
**Four**	2,372	71.4	Open dataset [12]	Patient ID
**Five**	2,357	71.1	Open dataset [12]	Patient ID
**Six**	2,369	71.6	Open dataset [12]	Patient ID
**Seven**	2,377	71.6	Open dataset [12]	Patient ID
**Eight**	2,352	70.7	Open dataset [12]	Patient ID
**Nine**	2,364	71.3	Open dataset [12]	Patient ID
**Zero**	2,376	71.8	Open dataset [12]	Patient ID
**Cataract**	484	60.1	Recorded by the authors	Procedure
**Phacoemulsification**	606	84.0	Recorded by the authors	Procedure
**Intraocular lens**	462	58.3	Recorded by the authors	Procedure

^a^Researchers recorded the target words provided by the text-to-voice tools.

As a preprocessing step, to reduce the imbalance between the classes, we oversampled the recorded target words of *time-out*, *cataract*, *phacoemulsification*, and *intraocular lens*. However, we partitioned the word dataset such that the audio from the same recording did not straddle the training and validation dataset split [13]. During data presentation, a short-time Fourier transform was conducted to compute the spectrograms. Due to the addition of our recorded audio, the spectrogram parameters of MATLAB audio project were tuned to avoid calculation errors. Moreover, we performed data augmentation to build a robust, trained model. Data augmentation is a widely used approach to boost the generalization of deep learning models and prevent overfitting [14]. We augmented data with the help of oversampling by amplifying sound waves and resizing the mel-spectrograms. Using MATLAB, we randomly scaled input sound data within the range of [−20%, +20%], translated the mel-spectrogram by up to 10 frames forward or backward in time, and scaled the mel-spectrograms along the time axis within a range of [-20%, +20%]. The mixed sound *z* was generated using *z =* (1*-w*)*∙x + w∙y* where *x* is the audio of the original sample and *y* is the signal of the noise data. We randomly scaled the mixed noise weight *w* within the range of [0, 0.5]. The final data distribution diagram is presented in S2 Fig.

### Training algorithm

**Fig 2:**
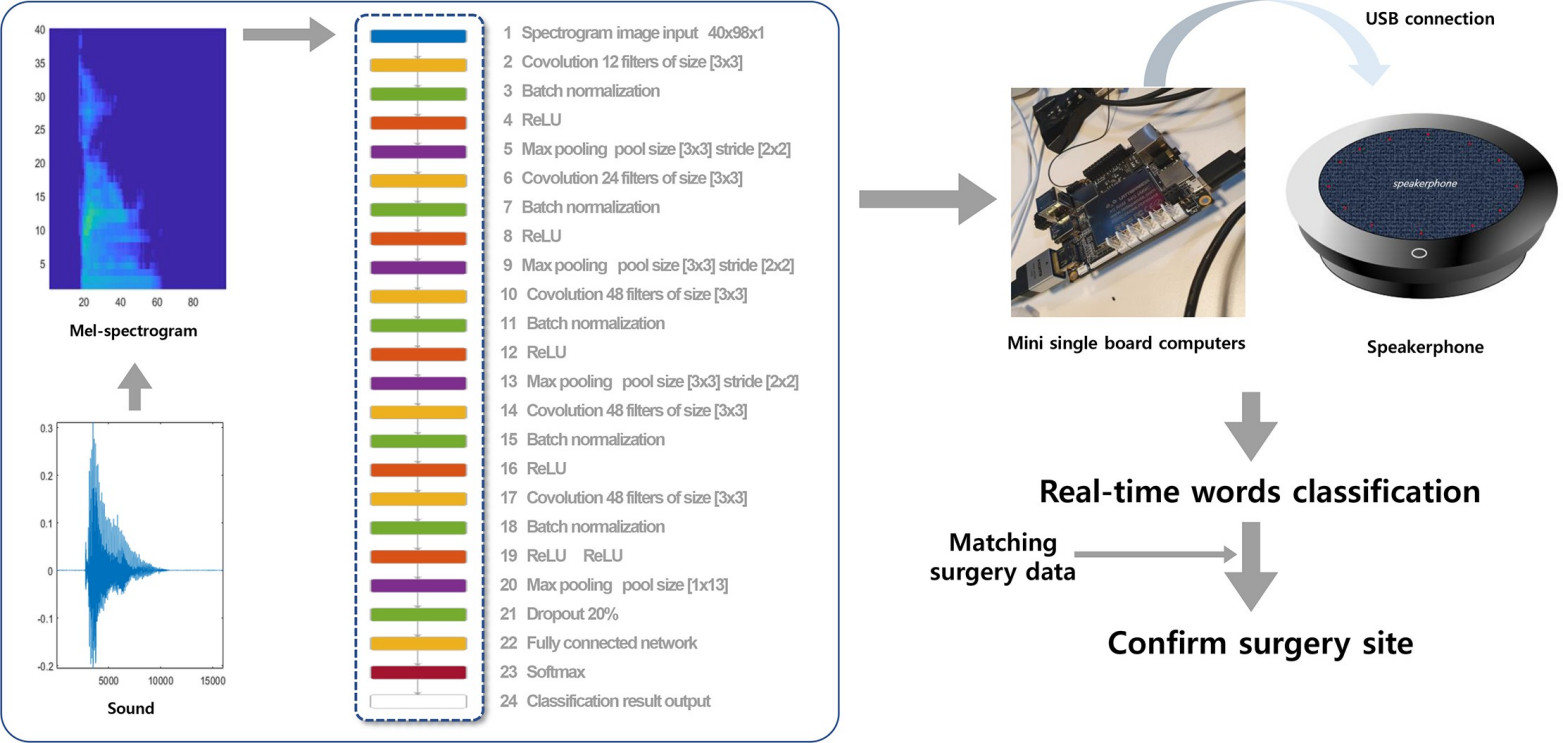
Deep learning architecture and application of LattePanda to build a smart speaker.

Initially, training and validation were conducted using MATLAB 2019a (Mathworks, Natick, MA, USA). The code for the deep learning model was based on MATLAB’s underlying audio processing project "Speech Command Recognition Using Deep Learning". The overview and detailed code of this project may be available at the official MATLAB website https://www.mathworks.com/help/deeplearning/examples/deep-learning-speech-recognition.html. These project codes assisted us in easily building a deep learning model for audio processing. We modified this speech recognition code to train a deep learning model for detecting the target words. Fig 2 represents the detailed framework of our deep learning and hardware model used to build a smart speaker. Mel-spectrograms were converted to 40-by-98-pixel images, and the deep learning model used them as input data. We used a deep convolutional neural network with 5 blocks that were composed of convolution layers, batch normalization, activation function (ReLU), and MaxPooling. To avoid overfitting of the deep learning model, the last fully connected layers were constrained via the drop-out technique (drop-out probability = 0.2). This architecture is widely used in CovNet-related models for audio event detection [15], and detailed layers and parameters are described in Fig 2. The training process was conducted using the Adam optimizer with a mini-batch size of 128 [16]. The initial learning rate was set at 0.0002, and the learning rate decayed every 20 epochs at an exponential rate of 0.1. The number of maximum epochs was set at 25 empirically. To detect the target words, the output threshold was set at 0.9. If the network output did not meet the threshold value, the word detection flag was not activated. The computer used in this study was equipped with NVIDIA GEFORCE GTX1060 3 GB GPU for transfer learning and with an Intel core i7 processor to train the deep learning models. After building the deep learning model, using a desktop computer, we implemented the smart speaker using LattePanda (LattePanda, Shanghai, China), which is a mini development single board computer [17], and Jabra Speakerphone 410 (Jabra GN, Portlabnd, Oregon, USA). MATLAB was installed in the mini board to run the trained deep learning model. The fully trained deep learning model in the desktop was copied to LattePanda and was operated under a MATLAB environment for the real-time experiments.

### Real-time experiments

The confirmation of a time-out requires accurate recognition of the keywords associated with the surgery and comparison between the recognized words and real surgical information. In this research, we assumed that the actual surgical information was input in the device before the operation. Once the surgical speech words were recognized, the system changed the flag variables linked to the patient’s identity and surgical site. In the case of an inconsistency between the recognized words (for example, left versus right), we chose the word that exhibited a higher probability as obtained by the deep learning model. The detailed codes for the real-time analysis are also presented in MATLAB’s underlying audio processing project.

To validate our model in consecutive speeches during time-outs, we generated the time-out script by randomly selecting the status of the surgical participants. The patient’s 5-digit identity numbers were generated randomly, and the left or right surgical site was also chosen randomly. The option for surgery was selected among cataract, vitrectomy, and intravitreal injection, which are the most common ophthalmic surgeries. Two types of time-out script are presented in S3 Fig, and one of them was selected randomly. During the experiments, we evaluated the accuracy of our deep learning model across multiple generated time-out speeches, using the smart speaker without a desktop computer. The time-out speeches were recorded based on the generated script by text-to-speech tools. We played 200 time-out audio clips and compared the recognized words and surgical information in the time-out script. If the device correctly recognized all instances of the word “*time-out*”, the patient’s 5-digit identity number, surgical site, and whether it was a cataract surgery or not, the audio was classified as a correct case. Because cataract surgery could be referred to in various synonyms, we categorized the audio as a cataract surgery case if the audio had at least one of the words “*cataract*”, “*phacoemulsification*”, and “*intraocular lens*”. In additional experiments using the web-based sources, the speech audios were streamed using the speaker of Samsung Galaxy 10. The experiments were performed at three different distances (0.5 m, 1.0 m and 1.5 m) between audio source and speakerphone to explore the effect of distance. To perform a comparative study using another device, we also adopted an additional speakerphone, which is a part of the Kakao Mini-C (KAKAO Corp., Jeju, South Korea).

## Results

**Fig 3:**
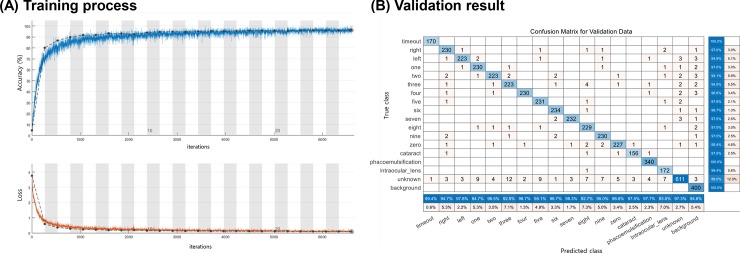
Training and validation results. (A) Learning Curves of the Deep Learning Model. (B) Confusion Matrix to present Classification Results for Validation Dataset.

We successfully trained the deep learning model by using the training dataset after the data augmentation. In total, 33,819 short-word audio clips were used for the training and 4778 clips were used for the validation of the model. Fig 3 demonstrates the training process and validation result. After 6600 training iterations (25 epochs) of the deep learning model, the training process was stopped to avoid over-fitting. The time taken to complete the training was 61 min. The confusion matrix presented in Fig 3 indicates that the total accuracy was 96.3% for the validation set. An example of several target words being detected by the deep learning model is demonstrated in S1 Video.

We found that our deep learning architecture and training epochs were appropriate to enable the best performance (Table 2). The deep learning model without batch normalization and that without dropout performed weaker than our network model. According to this result, the final deep learning model having batch normalization and drop-out layers with 25 training epochs was not over-parameterized.

**Table 2:** Validation accuracy according to different deep learning architectures and training epochs.

Models	Epochs	Accuracy (%)
Deep learning (CNN)	10	92.5
	25	96.3
	50	96.1
Deep learning (CNN without batch normalization)	10	88.2
	25	93.5
	50	94.0
Deep learning (CNN without dropout)	10	92.1
	25	95.5
	50	92.9

CNN, Convolutional neural network

Additional binary classification tests were performed to explore the robustness and performance of detection using test dataset (Table 3). Binary classification indexes were calculated using the output probability ratio of the two target words. We observed that the deep learning model outperformed random forest and support vector machine. In the “*left*” versus “*right*” problem, the sensitivity and specificity were 96.8% and 97.7%, respectively. When we tried to classify “*three*” from “*tree*,” which was included in the “*unknown*” class, the performance showed a sensitivity of 95.8% and specificity of 94.8%. Although it is a common pair of mispronounced words, we found that the classification performance was reliable.

**Table 3:** Binary classification results to explore the robustness and outcome of detection using the test dataset.

	AUC	Accuracy (%)	Sensitivity (%)	Specificity (%)
Problem 1: “*Left*” versus “*Right*” (*Index* = *P*_*left*_*/P*_*right*_)				
Deep learning (CNN)	0.996	97.3	96.8	97.7
Random forest	0.991	95.7	96.4	94.9
SVM using RBF kernel	0.978	93.1	91.8	94.3
Problem 2: “*Three*” versus “*Tree*” (*Index* = *P*_*three*_*/P*_*unknown*_)				
Deep learning (CNN)	0.988	95.3	95.8	94.8
Random forest	0.896	82.4	87.0	77.7
SVM using RBF kernel	0.885	83.6	82.0	85.1
Problem 3: “*Time-out*” versus “*Unknown*” (*Index* = *P*_*time-out*_*/P*_*unknown*_)				
Deep learning (CNN)	0.990	95.1	93.6	96.6
Random forest	0.980	92.9	95.4	90.3
SVM using RBF kernel	0.983	93.5	90.9	96.0

AUC, area under the receiver operating characteristic curve; RBF, radial basis function; SVM, support vector machine.

**Fig 4:**
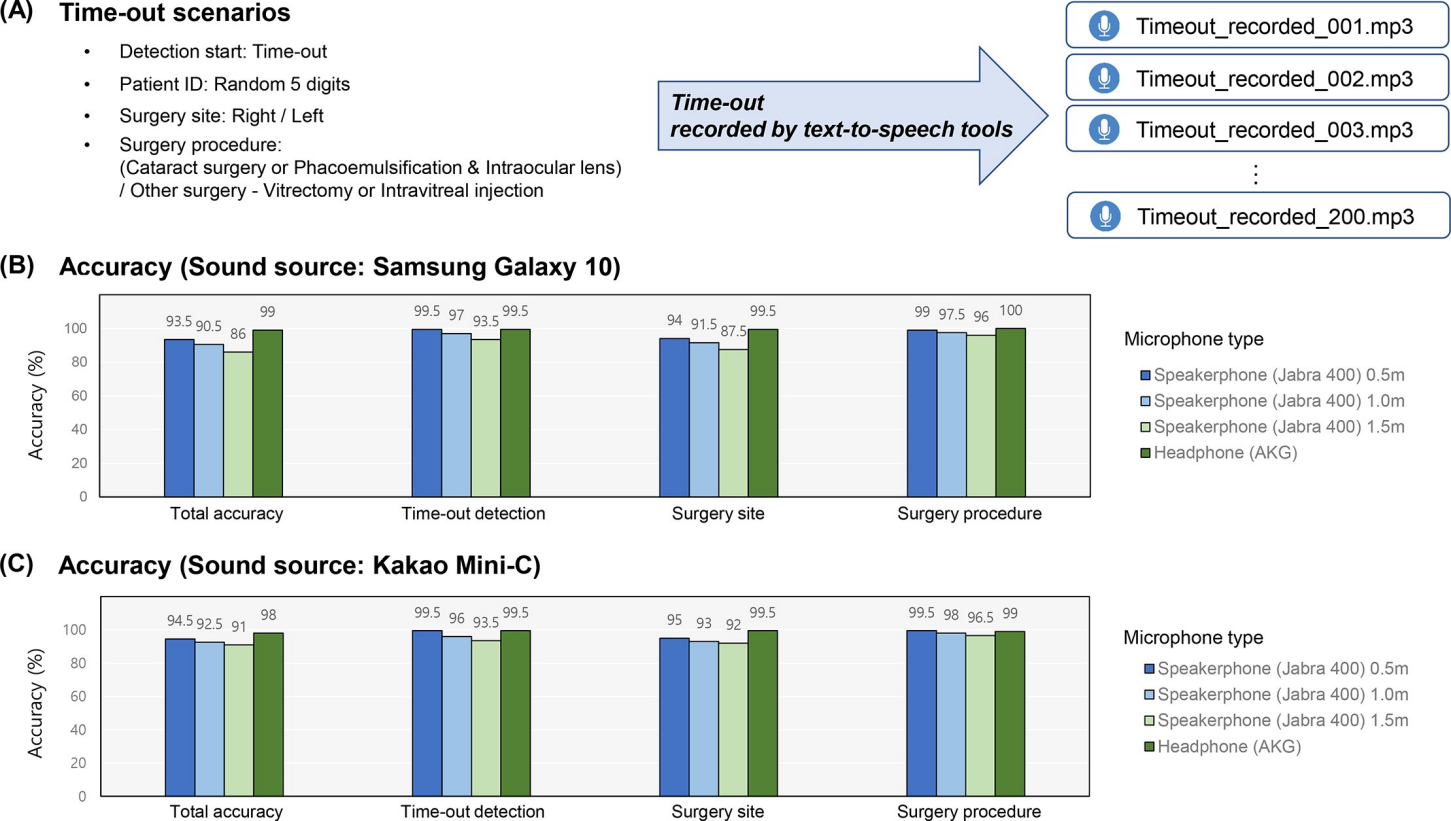
The real-time experiment using our developed smart speaker. (A) We generated the time-out script by selecting surgical status randomly. (B) The accuracy of the deep learning model using Samsung Galaxy 10 as a sound source. (C) The accuracy of the deep learning model using Kakao Mini-C as a sound source.

The real-time experiment, using our developed smart speaker, is presented in Fig 4. The surgical information and script were randomly generated for each trial. The deep learning approach was applied to 200 time-out audio clips recorded by text-to-speech tools, as described in the previous section. The final results of the deep learning model indicate a robust and good detection of the target words in the controlled setting. When we adopted Samsung Galaxy 10 as a sound source, the deep learning-based smart speaker achieved an accuracy of 93.5% with 0.5-m distance between the sound source and Jabra speakerphone. The accuracy of detecting patient’s surgical site was 94%, and it was the lowest compared to the accuracy of detecting the remaining surgical information. In this experiment, the accuracy of detection of the surgical site and procedure was 100%. We observed that the sound detection performance decreased with a longer distance of 1.5 m. The highest accuracy of 99.0% was observed with AKG headphones. When Kakao Mini-C was used as a sound source, the accuracy was 94.5% at a distance of 0.5 m, and the results were similar to those of the experiment with Samsung Galaxy 10.

**Fig 5:**
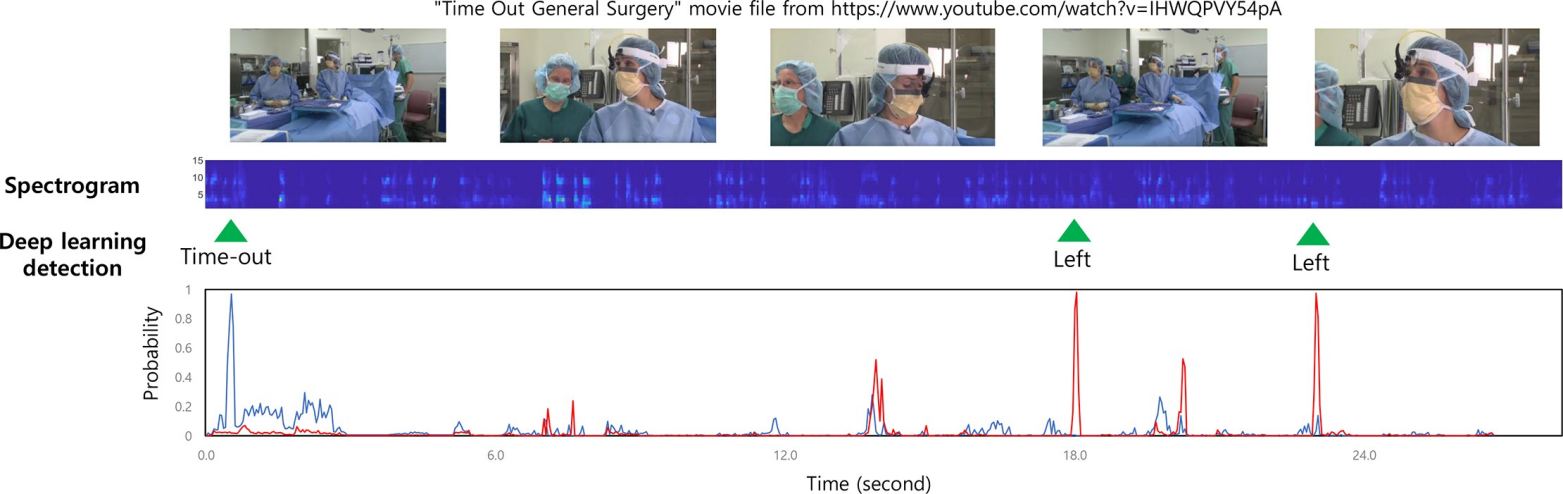
Example of the developed smart speaker using a video from YouTube.

The application of the deep learning model was not limited to the author’s voice dataset. Additionally, we also validated our deep learning model by using the time-out speech generated by the web translator sourced from https://papago.naver.com (S2 Video). Our model was successfully able to initialize the detection process on the time-out sign, identify the patient identity number, and confirm the surgical site and procedure. Another example, using a video clip from YouTube, is presented in Fig 5. In this case, for a non-ophthalmologic surgery (as shown in the video), we found that the deep learning model could only detect the time-out sign and surgical site. By considering the probabilities of the target words, the deep learning model was able to accurately classify the real-time input speech data.

## Discussion

This research deals with a challenging audio processing task that arises in operation rooms for cataract surgeries. Adopting the smart speaker-assisted time-out phase will improve the patients’ safety during cataract surgeries, especially considering the risk due to wrong-site surgeries. To the best of our knowledge, this proposed work is the first trial to develop a smart speaker for use in operation rooms. We believe that this work presents the first step forward in the development of smart operation rooms and will guide the following research associated with medical smart speakers. However, the accuracy was dependent on the distance and type of device. The performance of speech recognition should be further improved.

The smart speaker presented in this study can assist in the surgical timeout under reliable surveillance. A previous study highlighted that surgical safety checklists for every case can improve surgical outcomes [3]. This device can act as a safety monitor to confirm whether the time-out for surgical checklist was conducted before surgery [18]. If the surgeon and participants do not perform a time-out before the surgery, our device may caution them against neglecting the time-out. Thus, the smart operation room system that uses this device can significantly contribute to reducing human errors and increasing the safety of patients. This framework can extend to the confirmation of an intraocular lens in cataract surgeries or ablation depth in corneal refractive surgeries. As the selection of the incorrect power of an intraocular lens is reported as the most common error in ophthalmic surgery, future studies should consider including the lens information during a time-out [6]. Additionally, displaying visual information on screen about surgery along with audio information will improve the confirmation during time-out. We believe that a smart operation room will be able to combine information from various sources, and audio will play an important role in reducing the occurrence of wrong site surgery.

With advances in digital health technologies, the smart speaker will revolutionize medical fields. The major features of the smart device system include a connection to the network, ubiquity, embedded intelligence, and programmability [19]. A previous study suggested that the smart system using voice-controlled technology presents new opportunities for the care of diabetic patients having complications in their lower extremities [20]. Considering the aseptic conditions during an interventional radiology procedure, a machine learning smart speaker was developed to provide device information to the clinicians [11]. A more comprehensive study, using a smart speaker, was performed to predict cardiac arrests by using real-world 9-1-1 audio [13]. As the presence of agonal breathing is associated with cardiac arrest, this study attempted for the prediction of cardiac arrest by using the support vector machine technique. Our study focused on the surgical time-out to reduce medical errors and improve patients’ safety. Thus, in the near future, an artificial intelligence-based smart speaker might lead to major changes in medical fields.

Deep learning could play an important in directly predicting the words to confirm the patient’s information. In a previous study, spectrograms and a binary classification model combining a deep learning feature extractor and support vector machine, were used [13]. Similarly, in our study, mel-spectrograms and a multi-categorical classification model to detect various target speeches, were used, with the help of deep learning. Although the number of classes was 18, the detection accuracy was sufficient to justify applying the trained model for real-time word detection. Our deep learning architecture was based on CovNet-related model for audio event detection [15], and it was unable to consider the semantic relationship between words As our work did not utilize natural language processing (NLP) or recurrent neural network (RNN), there is a lot of potential to improve the performance. RNN is highly efficient in predicting sequential data due to dependency building in neighboring words [21]. Therefore, a more comprehensive recognition for a time-out might be conducted by combining our deep learning model and RNN.

Recent development of open access deep learning techniques, publicly available audio vocabulary, and lightweight hardware allows us to conveniently build a smart speaker. In our experience, it was not difficult to implement a deep learning model in a small-sized development board such as LattePanda and Raspberry series, and these boards are easily accessible. Especially, the AIY Voice Kit released by Google in 2017, is an inexpensive and powerful tool that can be used to build a smart speaker [22]. This device is based on Raspberry. Therefore, it can be programmed even with minimal Python coding expertise. Moreover, we used LattePanda, which runs a full version of Windows 10 and MATLAB 2019a. Therefore, the trained deep learning model using MATLAB 2019a, could easily be imported into the device. As this device can be connected to a monitor screen via a high definition multimedia interface, the surgical information and time-out status can be visualized for surgeons and other participants. We believe that the deep learning-based small device will play a significant role in the future of smart operation rooms.

The current pilot study has several limitations. First, a number of voice datasets recorded by the authors produced fundamental limits. Most deep learning researchers agree that a small amount of data is insufficient to test the effectiveness [14]. We used audio data augmentation in order to overcome this challenge. In addition, there was an absence of an external validation dataset to confirm the performance of the classification models. Second, our sound dataset was obtained in a limited controlled condition, although various background noises were used. A key challenge to this system is accessing the real-world data of surgical time-outs. To overcome this challenge, in the future, real time-out voice data has to be collected from operation rooms. Third, our study showed that the patient’s data and surgical information has to be entered manually in this system prior to the time-out phase. In this pilot study, we focused on the accurate detection of target words and confirmation of the surgical site. To achieve a smart operation room system in a hospital, medical data should be automatically imported to the device. We are currently planning a further study to address these limitations and enable the model to automatically update the electronic medical records.

## Conclusion

Even though wrong-site surgeries are rare, they have been a serious problem in ophthalmology. Therefore, a new strategy that utilizes artificial intelligence techniques, which is automated, reliable, and low-cost, needs to be considered. In this pilot study, our deep learning-based smart speaker was able to successfully confirm surgical information during the time-out speech. Future studies should focus on collecting real-world time-out data and automatically connecting the device to electronic health records. By building a technologically advanced surgery room system to confirm the surgery site by using the deep learning-based smart speaker, future technologies can reduce the number of medical errors and improve the quality of life for patients as well as surgeons.

## Supporting information

S1 Fig Text-to-speech tools used to generate sounds of target words. (PDF)

S2 Fig Dataset distribution in the training and validation set. (PDF)

S3 Fig Two types of time-out script for the real-time experiment. (PDF)

S1 Video Examples of detection of target words recorded by an author. (MP4)

S2 Video A time-out example of confirmation of surgical information by the deep learning model. (MP4)
