# Supplementary material for: Deep learning-based smart speaker to confirm surgical sites for cataract surgeries: A pilot study
Source: PLoS One. 2020 Apr 9;15(4):e0231322. doi: 10.1371/journal.pone.0231322 (PMC7144990; doi:10.1371/journal.pone.0231322)
Supplement: S1 Fig — (PDF) [file pone.0231322.s001.pdf]

S1 Fig. Text-to-speech tools used to generate sounds of target words.

We trained and validated the generalization of our deep learning network in detecting target words that were not present in the Google The Speech Commands dataset. We recorded speaking with varying accents, speed, and voice tones that provided by the text-to-voice tools. These figures demonstrated the text-to-voice tools used to collecting sound data in this study.

a) TTSREADER (https://ttsreader.com/)

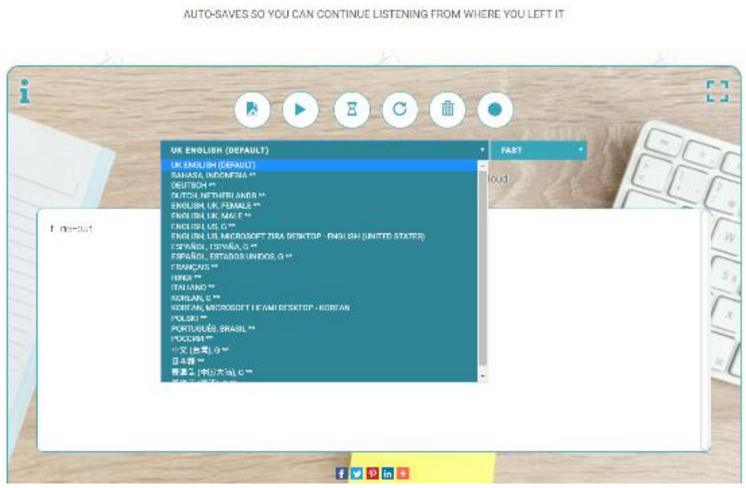

b) IBM Watson Text to Speech Demo (https://text-to-speech-demo.ng.bluemix.net/)

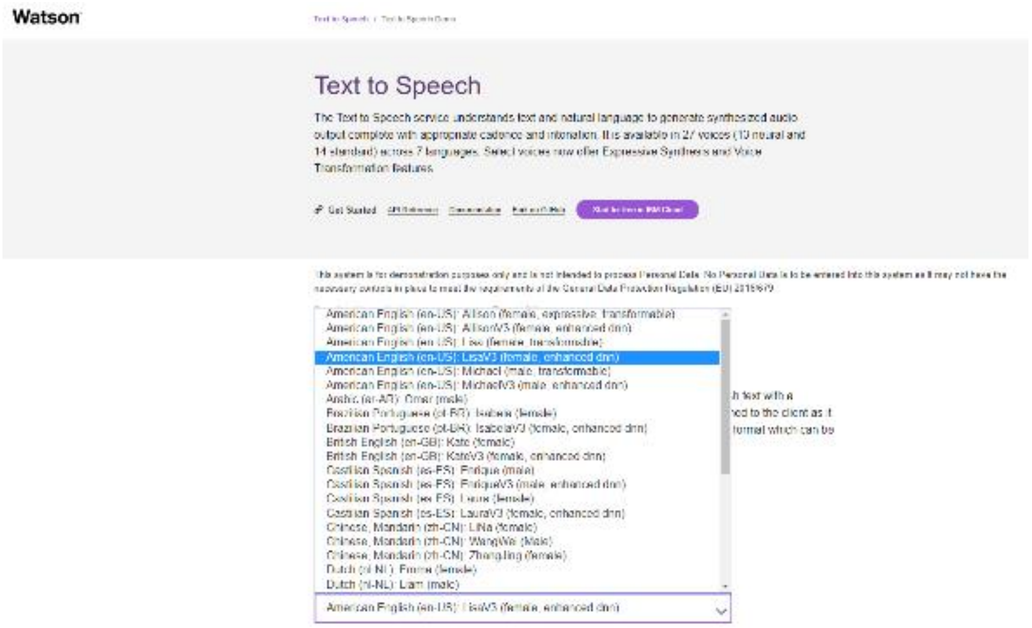

c) Nuance

(https://www.nuance.com/omni-channel-customer-engagement/voice-and-ivr/text-to-speech.html#!)

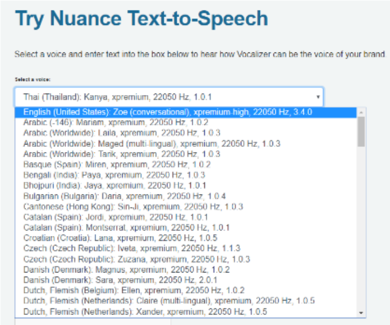

d) Google Cloud (https://cloud.google.com/text-to-speech)

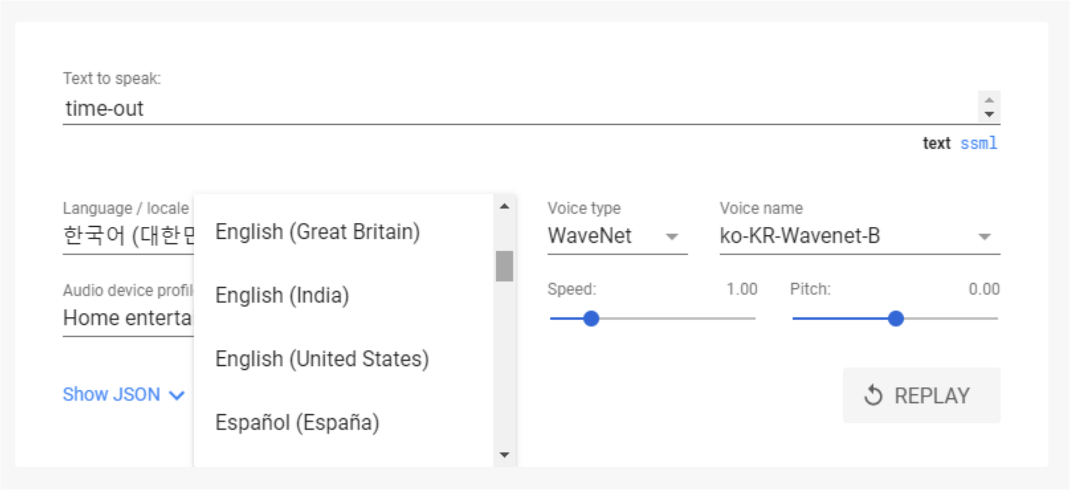

e) Vocalware (https://ttsdemo.com/)
