# Supplementary figures and images for: Deep learning-based smart speaker to confirm surgical sites for cataract surgeries: A pilot study
Source: PLoS One. 2020 Apr 9;15(4):e0231322. doi: 10.1371/journal.pone.0231322 (PMC7144990; doi:10.1371/journal.pone.0231322)

S2 Fig. Dataset distribution in the training and validation set.

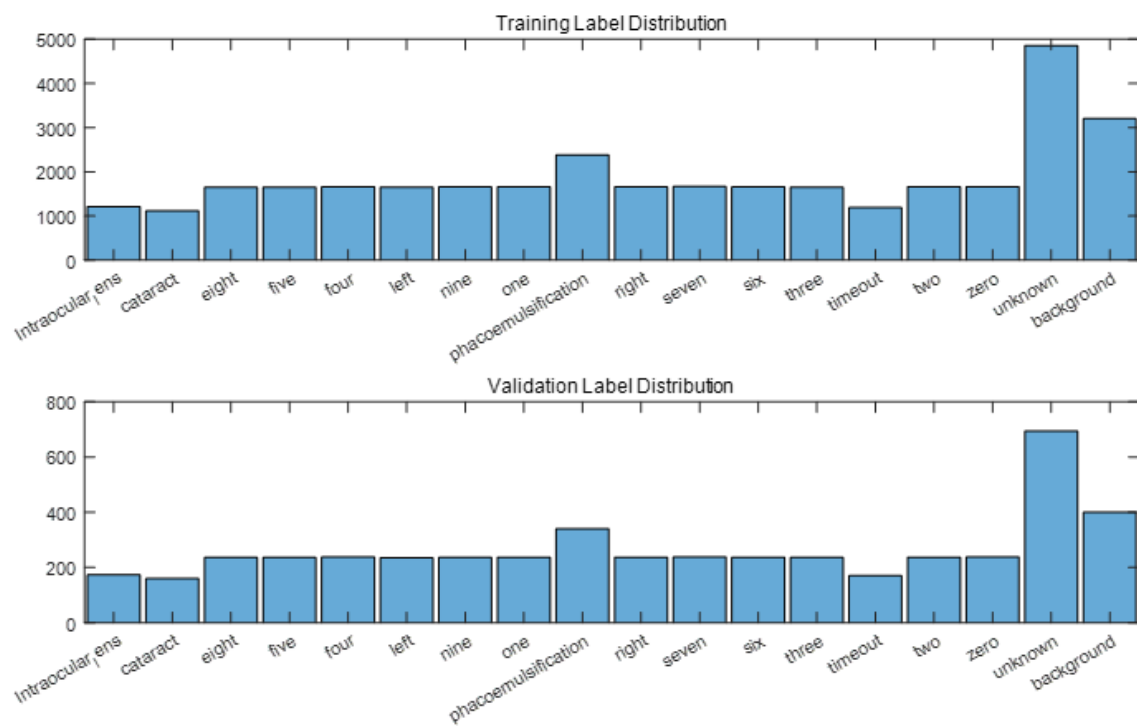

Supplement: S2 Fig — (PDF) [file pone.0231322.s002.pdf]
