# Supplementary material for: Deep learning-based smart speaker to confirm surgical sites for cataract surgeries: A pilot study
Source: PLoS One. 2020 Apr 9;15(4):e0231322. doi: 10.1371/journal.pone.0231322 (PMC7144990; doi:10.1371/journal.pone.0231322)
Supplement: S3 Fig — (PDF) [file pone.0231322.s003.pdf]

S3 Fig. Two types of time-out script for the real-time experiment.

- Type A

  Is everybody ready for time-out? The patient is Mrs. Kim, her ID is (one, one, three, five, seven). The surgery site is (right) eye. Is it correct? Yes, it is correct. This surgery consists of (phacoemulsification and intraocular lens implantation).

- Type B

  We are ready for time-out. First, we should check the patient. Mr. Park, his ID number is (two, nine, zero, zero, and seven.) He requires surgery on his (left) eye. He will undergo (cataract surgery) on his (left) eye. Is everything correct?
